# Supplementary material for: Repeated dosing improves oncolytic rhabdovirus therapy in mice via interactions with intravascular monocytes
Source: Commun Biol. 2022 Dec 19;5:1385. doi: 10.1038/s42003-022-04254-3 (PMC9761050; doi:10.1038/s42003-022-04254-3)
Supplement: Supplementary file 2 — Supplementary Information [file 42003_2022_4254_MOESM2_ESM.pdf]

Supplementary Information

Repeated dosing improves oncolytic rhabdovirus therapy in mice via interactions with intravascular monocytes

Victor Naumenko, Jahanara Rajwani, Madison Turk, Chunfen Zhang, Mandy Tse, Rachelle P. Davis, Daesun Kim, Andrea Rakic, Himika Dastidar, Shinia Van, Laura K. Mah, Esha K. Kaul, Vladimir P. Chekhonin, Douglas J. Mahoney, Craig N. Jenne

Supplementary Figures

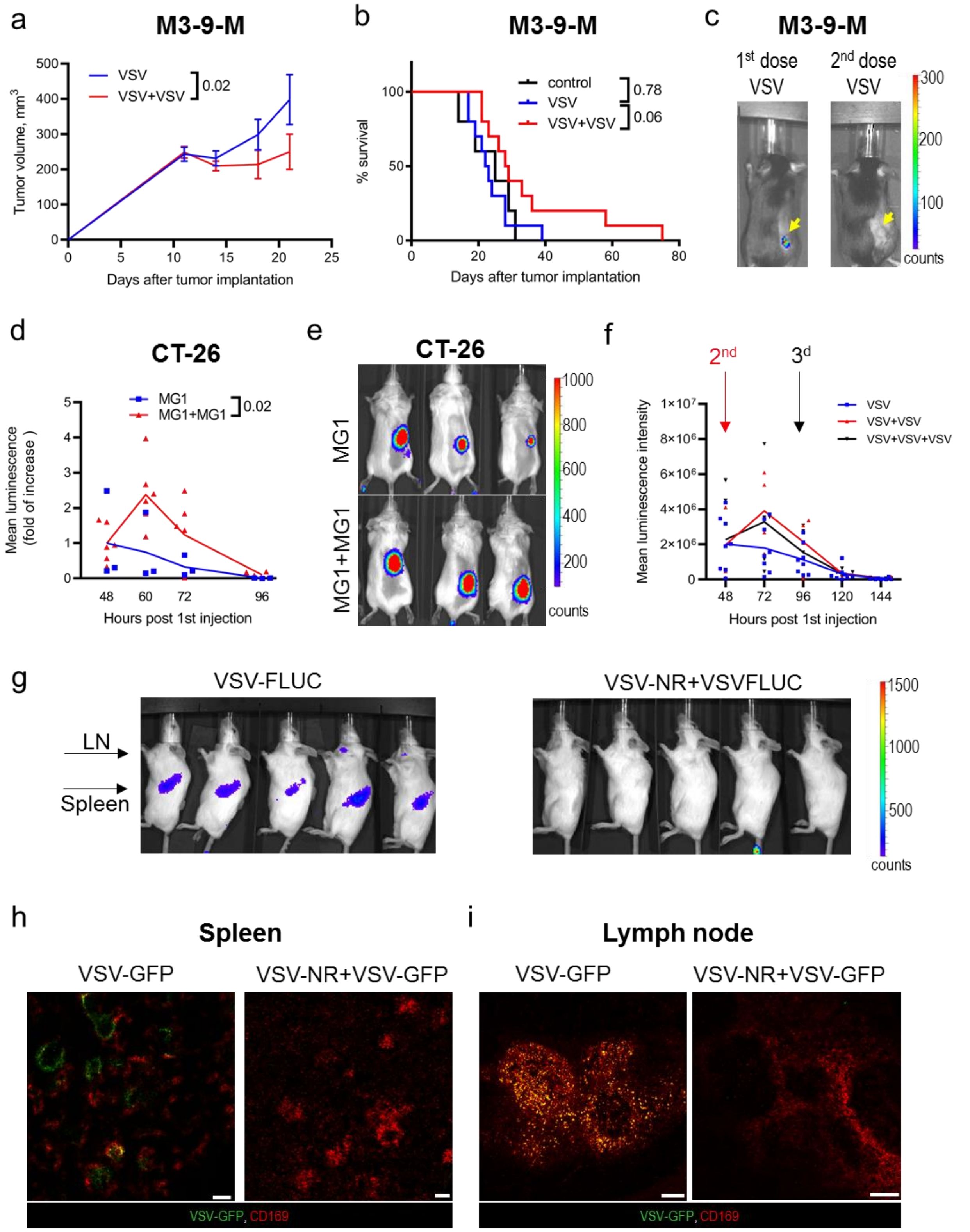

Supplementary Figure 1. OV delivered as a second dose does not infect the tumor yet enhances the infection of the initial OV treatment. a. Tumor measurements for M3-9-M-bearing mice injected with one or two doses of VSV (5×10^8 PFU, 48 h between i.v. injections, n=10). Results are shown as mean ±SEM; two-way ANOVA. **b.** Kaplan-Meier survival plots for the

groups shown in (**a**) and untreated tumor-bearing animals (n=5); log-rank test. **c.** Representative bioluminescence images of M3-9-M tumors (arrows) 24 h after first or second VSV-FLUC i.v. injection (5×10^8 PFU, 48 h between injections). **d.** Luminescence intensity for $CT26^{LacZ}$ tumors after a single (n=3) or repeated (n=6) MG1-FLUC dosing (5×10^8 PFU, 48 h between i.v. injections) Luminescence intensities for individual tumors are normalized to the mean luminescence intensity in each group at 48 h post first dose injection. Results are shown as mean with individual values; two-way ANOVA. **e.** Representative bioluminescence images for the groups shown in **(d)** at 72 h following a single MG1 dose or 24 h post second MG1 dose. **f.** Luminescence intensity for $CT26^{LacZ}$ tumors after treatment with one (n=8), two (n=4) or three (n=5) VSV doses (first dose – VSV-FLUC, repeated doses – VSV-GFP; 48 h between consequent i.v. injections). Results are shown as mean with individual values. **g.** Representative bioluminescence images of $CT26^{LacZ}$-bearing mice at 8 h post first or second VSV-FLUC i.v. injection (10^6 PFU, 48 h between injections). Representative confocal images of the spleen **(h)** and inguinal lymph node **(i)** at 8 h post first or second VSV-GFP i.v. injection (5×10^8 PFU, 48 h between injections). Infected cells are detected by GFP-transgene expression (green). Metallophilic macrophages in spleen **(h)** and subcapsular sinus macrophages in lymph nodes **(i)** are stained by anti-CD169 antibodies (red). Scale bar, 200 µm.

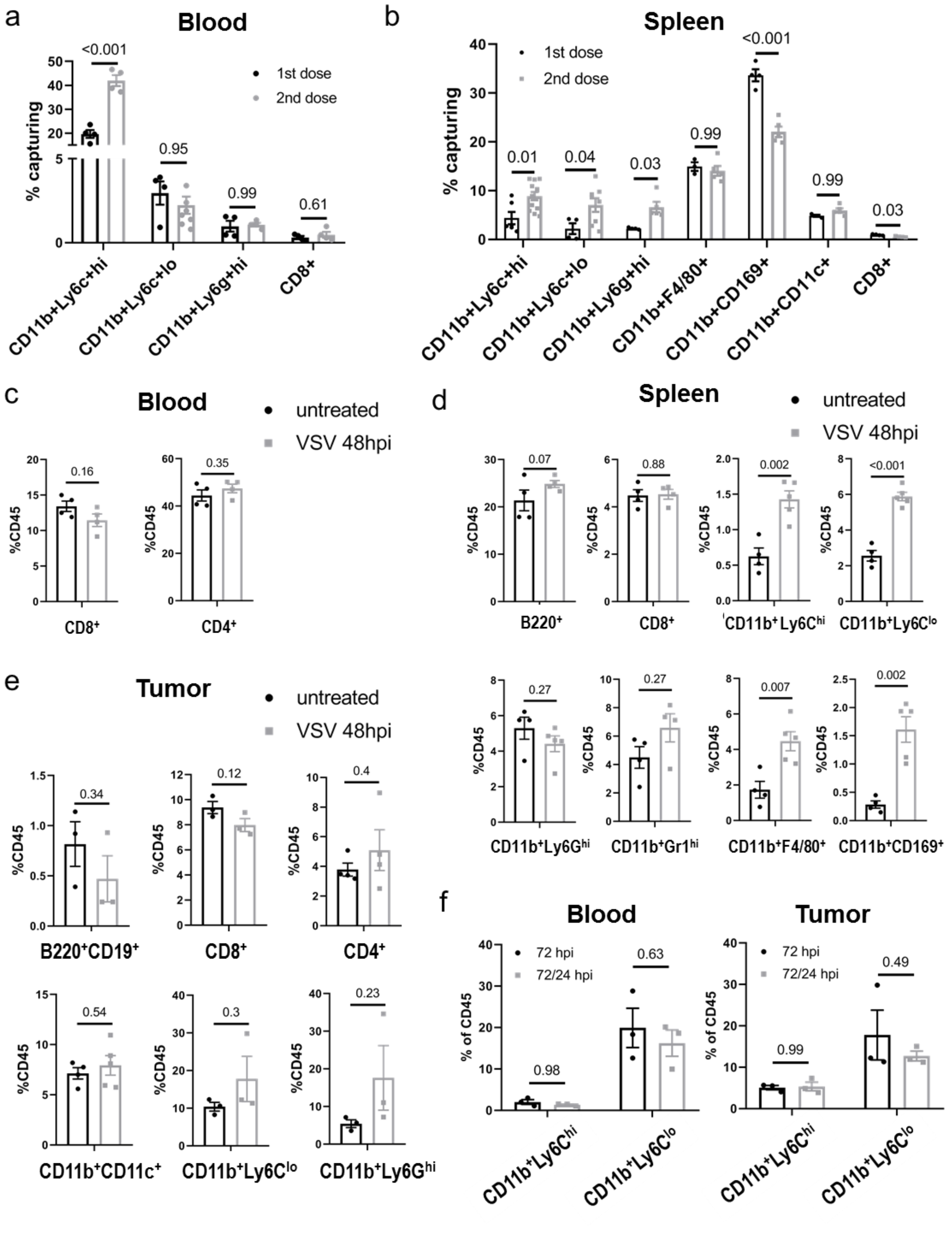

Supplementary Figure 2. Intravascular leukocytes recruited to OV-infected tumors interact with OV administered during a second dose. a. Flow cytometric analysis of blood cells 30 minutes after first or second VSV-AF647 i.v. injection. Results are shown as percentage of VSV-bound cells and are plotted as mean ±SEM (n=4); unpaired t-test. **b.** Flow cytometric analysis of spleen cells 30 minutes after first or second VSV-AF647 i.v. injection. Results are shown as percentage of VSV-bound cells and are plotted as mean ±SEM (n=4); unpaired t-test. **c-e.**

Peripheral blood **(c)**, spleens **(d)**, and tumors **(e)** from untreated mice or 48 h post i.v. injection of VSV (10^6 PFU) were analyzed by flow cytometry (Associated with **Figure 2k, l**). Results are shown as percentage of CD45+ cells and are plotted as mean ±SEM (n=4 **(c, d)**; n=3**(e)**); unpaired t-test. **f.** FC analysis of blood and tumor samples collected 72 h following a single VSV dose or 24 h post second VSV dose. Results are shown as percentage of CD45+ cells and plotted as mean ±SEM (n=3, unpaired t-test).

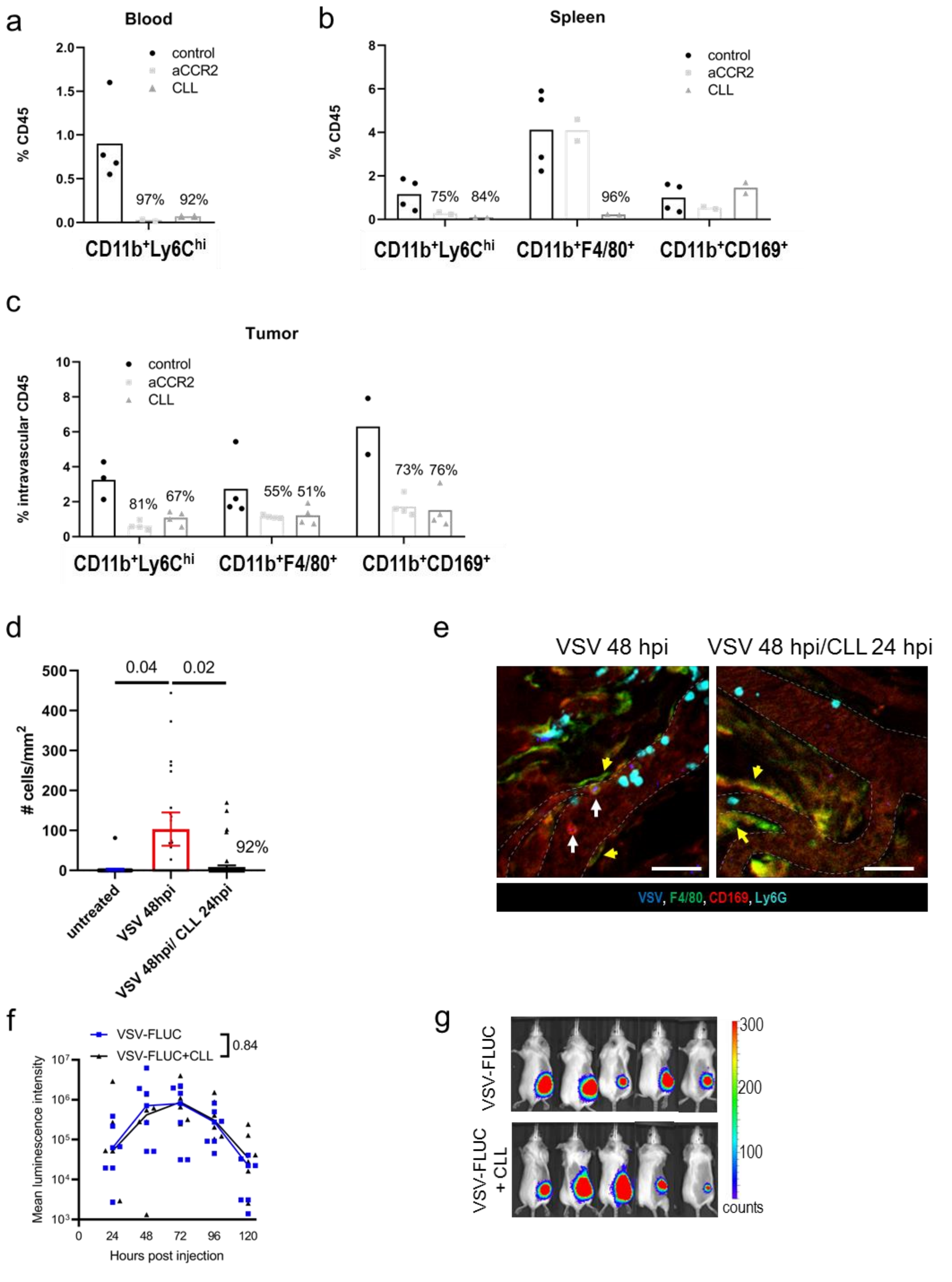

Supplementary Figure 3. Interactions between OV and inflammatory monocytes promote better infection of the initial OV dose. Flow cytometric analysis of monocytes/macrophages in blood **(a)** and spleen **(b)** 24 h post 20 µg anti-CCR2 i.p. injection or 1 mg CLL i.v. injection. Results are shown as percentage of CD45+ cells and plotted as mean with individual values.

Depletion efficiencies (%) are indicated on graphs. **c.** Flow cytometric analysis of intravascular monocytes in tumor samples 24 h post 20 µg anti-CCR2 i.p. injection or 1 mg CLL i.v. injection. Intravascular fraction of leukocytes is identified by anti-CD45 injected into the tail vein 10 minutes before animal euthanasia. Results are shown as percentage of intravascular CD45+ cells and plotted as mean with individual values. Depletion efficiencies (%) are indicated on graph. **d.** IVM analysis of monocytes (intravascular CD169+F4/80+ cells) in CT26^{LacZ} tumors of untreated animals or 48 h following i.v. administration of VSV (10^6 PFU) with or without CLL administered i.v. 24 h before imaging. Results are shown as cells per mm^2 and are plotted as mean ±SEM; one-way ANOVA followed by Tukey's multiple comparisons test. Depletion efficiency (%) is indicated on graph. **e.** Representative images of CT26^{LacZ} tumors 48 h post VSV i.v. injection (10^6 PFU) with or without CLL. Images are captured immediately following i.v. injection of VSV-AF647 (blue) as a second dose. White arrows show monocytes (intravascular cells expressing CD169 (red) and F4/80 (green)); yellow arrows indicate macrophages (interstitial CD169+F4/80+ cells). CLL injection depletes monocytes while tumor macrophages remain unaffected. Tumor vessels are delineated by white dashed lines. Cyan, neutrophils. Scale bar, 50 µm. **f.** Luminescence intensity for CT26 tumors following a single OVT treatment (10^6 PFU VSV-FLUC i.v.) with or without CLL treatment 24 h post virus administration. Results are shown as mean with individual values (n=6); two-way ANOVA. **g.** Representative bioluminescence images for the groups shown in **(f)** at 72 h post virus injection.

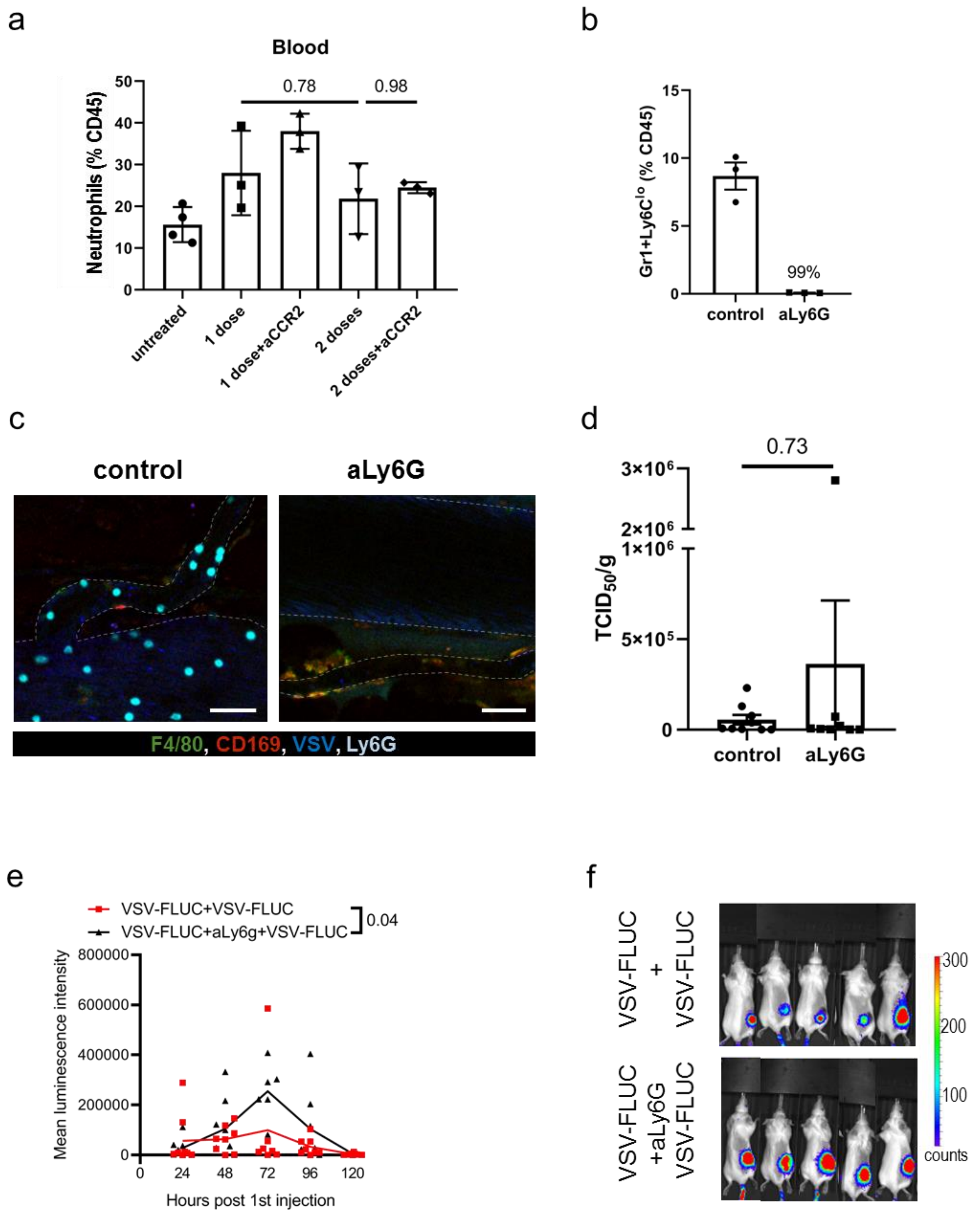

Supplementary Figure 4. OVT limits, in a monocyte-depended fashion, neutrophil antiviral activity within infected tumors. a. Neutrophil counts in blood samples collected from untreated mice or 72 h following a single VSV dose or 24 h post second VSV dose (10^6 PFU) ± 20µg anti-CCR2 treatment 24 h after initial virus administration. Results are shown as a percentage of CD45+ cells and are plotted as mean ±SEM (n=3); one-way ANOVA followed by Tukey's multiple comparisons test. **b.** FC analysis of neutrophils in CT26^{LacZ} tumor 24 h post 250 µg

anti-Ly6G i.p. injection. Results are shown as mean ±SEM (n=3); depletion efficiency (%) is indicated on graph. **c.** Representative IVM images of tumor vessels (delineated by white dashed-lines) in untreated and neutrophil depleted mice 24 h post 250 µg anti-Ly6g i.p. administration. Cyan, neutrophils; green, F4/80; red, CD169; blue, VSV-AF647. Scale bar, 50 µm. **d.** Virus titers in CT26LacZ tumors at 72 h post single dose treatment in animals with or without Ly6G depletion (n=9). Results are shown as mean ±SEM; Mann–Whitney test. **e.** Luminescence intensity for $CT26^{LacZ}$ tumors following 2 doses of VSV-FLUC (10^6 PFU, 48 h between i.v. injections) ± neutrophil depletion by 250 µg anti-Ly6G i.p. injection 24 h after first virus administration. Results are shown as mean with individual values (n=8); two-way ANOVA. **f.** Representative bioluminescent images for groups shown in **(e)** 24 h post second virus injection.

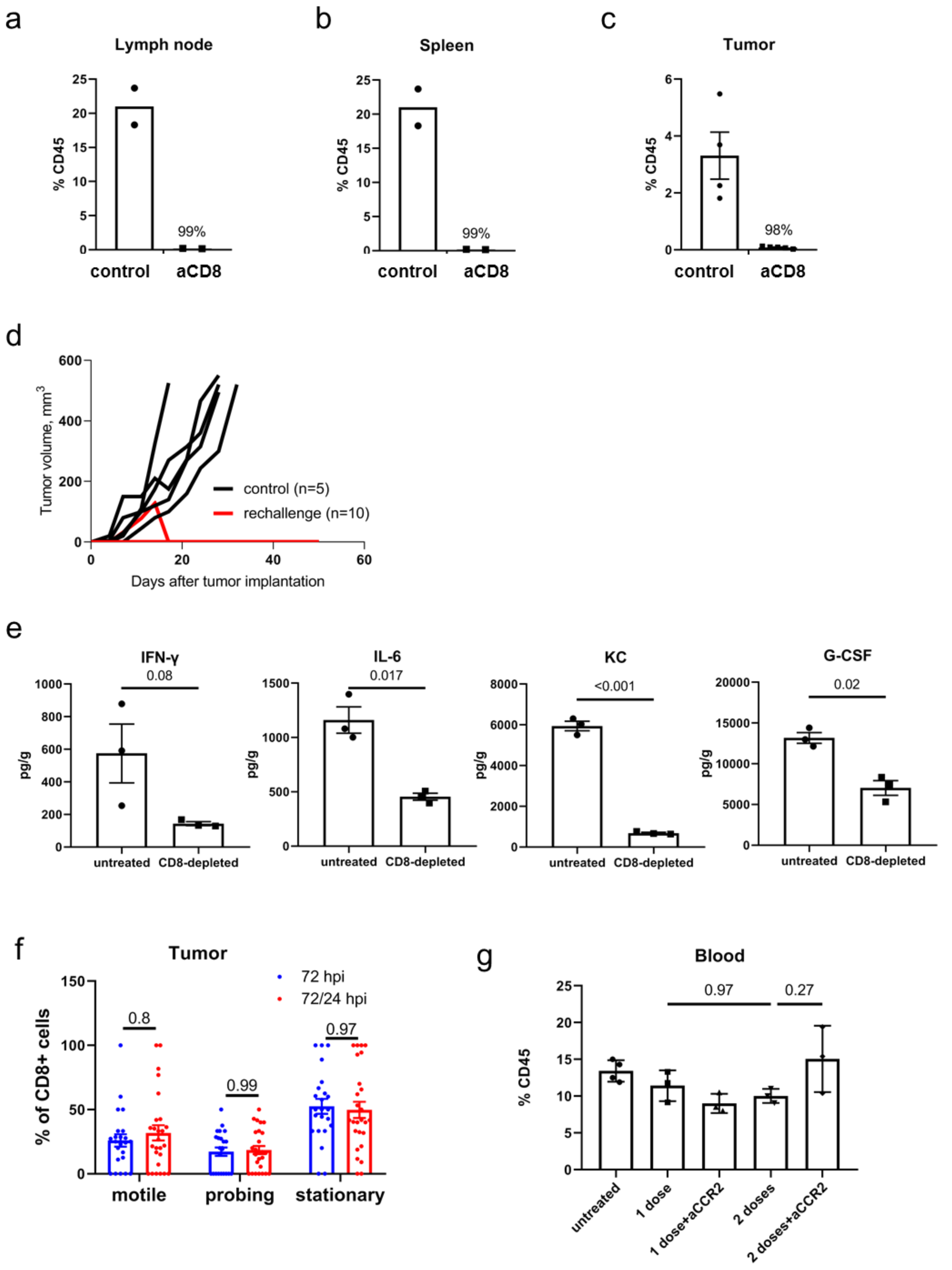

Supplementary Figure 5. OV-monocyte interactions promote CD8+ T cell recruitment. a-c. Flow cytometric analysis of CD8+ cells from the inguinal lymph node **(a)**, spleen **(b)**, and tumor **(c)** following depletion by i.p. administration of anti-CD8a (clone YTS 169.4) monoclonal antibodies. Anti-CD8b antibodies (clone YTS156.7.7) are used to detect CD8+ cells by flow cytometry. Results are shown as a percentage of CD45+ cells and are plotted as mean with

individual values (**a, b,** n=2) or mean ±SEM (**c**, n=4). Depletion efficiencies (%) are indicated on graphs. **d.** Animals (n=10) that had cleared $CT26^{LacZ}$ tumors upon VSV treatment were rechallenged with 10^6 $CT26^{LacZ}$ cells (s.c.) on day 100±10 after original tumor implantation. Naïve age-matched mice (control, n=5) received the same s.c. implantation dose. Individual tumor growth curves are plotted. **e.** Concentration of IFN-γ, IL-6, KC, G-CSF in tumor interstitial fluid from animals with or without CD8 depletion. Results are shown as mean ±SEM (n=3); unpaired t-test. **f.** IVM analysis of CD8+ cell behavior 72 h following a single VSV dose or 24 h post second VSV dose; mean ±SEM (two-way ANOVA followed by Sidak's multiple comparisons test). **g.** CD8+ T cell counts in blood samples collected from untreated mice or 72 h following a single VSV dose or 24 h post second VSV dose (10^6 PFU) ± 20 µg anti-CCR2 i.p. 24 h after initial virus treatment. Results are shown as a percentage of CD45+ cells and are plotted as mean ±SEM (n=3); one-way ANOVA followed by Tukey's multiple comparisons test.

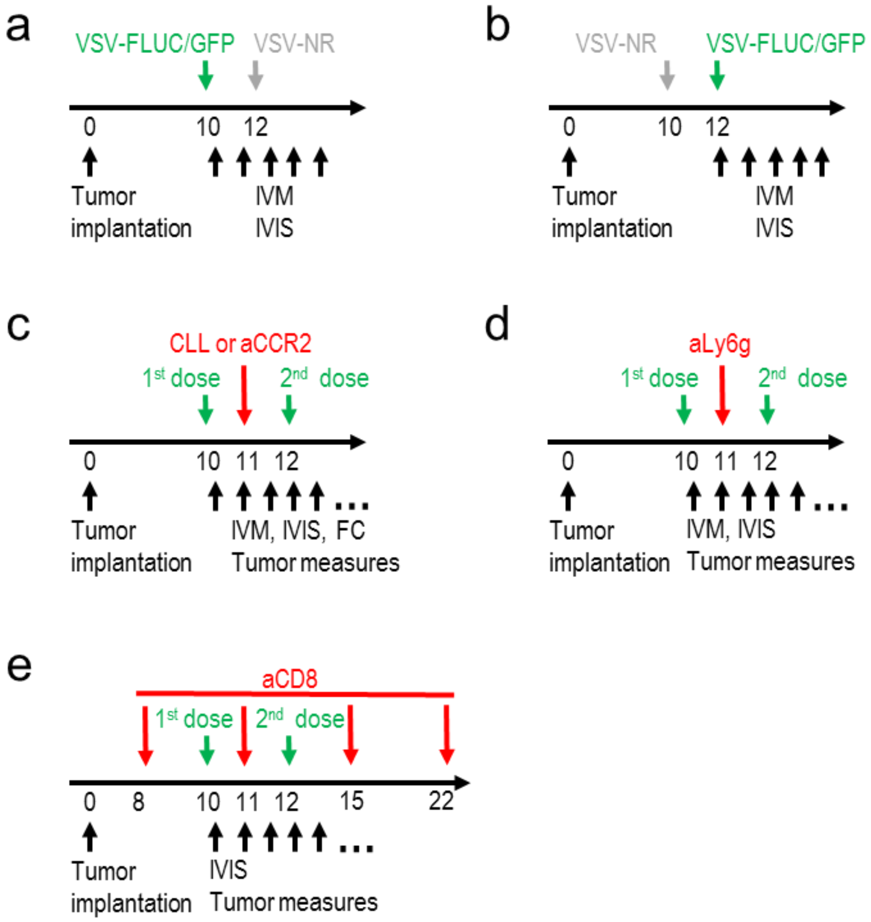

Supplementary Figure 6. Experimental schedules. Syngeneic subcutaneous tumors were established by subcutaneous injection of 10^6 $CT26^{LacZ}$ or $CT26^{LacZ}$ -RFP cells. On Day 10 mice were treated with one dose of $VSV^{\Delta M51}$ (10^6 PFU) with or without a second dose 48 h after initial treatment. **a.** To determine the specific contribution of the first dose animals were given with $VSV^{\Delta M51}$-FLUC/GFP followed by the second injection of $VSV^{\Delta M51}$-NR. **b.** To assess the contribution of the second dose $VSV^{\Delta M51}$-NR was administered first followed by $VSV^{\Delta M51}$-FLUC/GFP. **c**. Monocyte depletion was achieved following i.p. administration of 1 mg/kg of anti-CCR2-specific monoclonal antibodies (MC-21) or i.v. administration of 50 mg/kg clodronate liposomes (CLL). **d.** For neutrophil depletion, animals were treated with 10 mg/kg anti-Ly6G (1A8)-specific antibodies. **e.** CD8+ T cell depletion was achieved with anti-CD8 specific antibodies (clone YTS 169.4; 12.5 mg/kg on day 8 after tumor implantation followed by injections of 5 mg/kg i.p. on days 11, 15, 22).

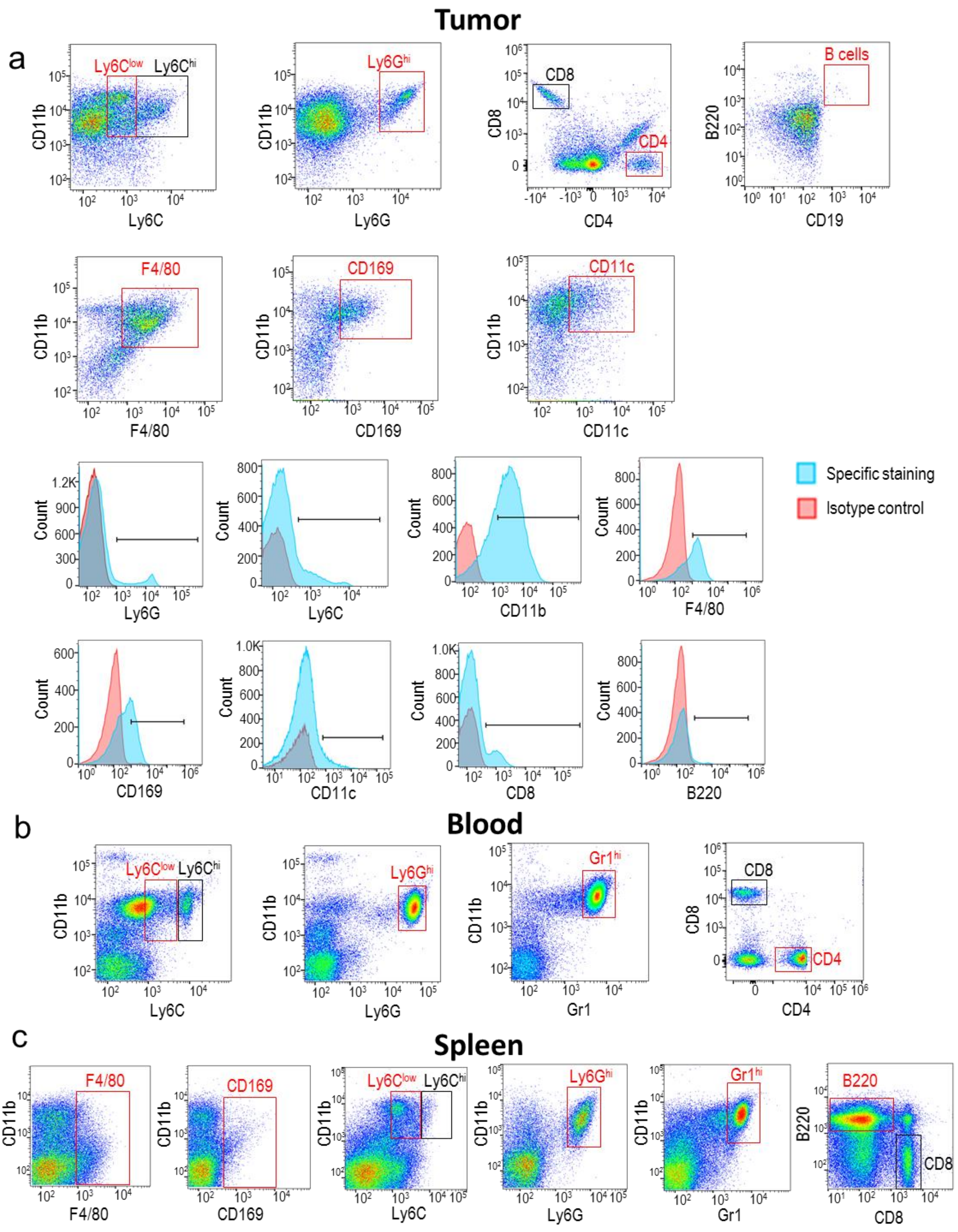

Supplementary Figure 7. Flow cytometry gating strategy. a-c. Identification of leukocyte subsets in tumor (**a**), blood (**b**) and spleen (**c**).
