# Supplementary material for: Repeated dosing improves oncolytic rhabdovirus therapy in mice via interactions with intravascular monocytes
Source: Commun Biol. 2022 Dec 19;5:1385. doi: 10.1038/s42003-022-04254-3 (PMC9761050; doi:10.1038/s42003-022-04254-3)
Supplement: Supplementary file 3 — Description of Additional Supplementary Files [file 42003_2022_4254_MOESM3_ESM.pdf]

Description of Additional Supplementary Files

File name: Supplementary Data 1

Description: The source data behind the graphs in the paper.

File name: Supplementary Movie 1

Description: First injection: VSV binds to tumor endothelium (part 1) and neutrophils (part 2). Blue, VSV- AF647; green, Ly6g; gray, vessels (FITC-BSA); red – CD31 (part 1) or tumor cells (part 2).

File name: Supplementary Movie 2

Description: Second injection: VSV is mainly captured by intravascular tumor monocytes identified by CD11b (part 1), Ly6c (part 2) and CD169 (part 3) expression. Blue, VSV-AF647; green, Ly6g; red – CD11b (part 1), Ly6c (part 2) or CD169(part 3).

File name: Supplementary Movie 3

Description: Neutrophils migrate to the spots of VSV infection in tumor. Green, VSV-GFP; magenta, Ly6g; gray, vessels (FITC-BSA).

File name: Supplementary Movie 4

Description: Longlasting interactions of virus-bound monocytes with neutrophils in CT26 tumors. Blue, VSVAF647; green, Ly6g; red, CD11b
