# Supplementary material for: Repeated dosing improves oncolytic rhabdovirus therapy in mice via interactions with intravascular monocytes
Source: Commun Biol. 2022 Dec 19;5:1385. doi: 10.1038/s42003-022-04254-3 (PMC9761050; doi:10.1038/s42003-022-04254-3)
Supplement: Supplementary file 9 — Reporting Summary [file 42003_2022_4254_MOESM9_ESM.pdf]

nature portfolio

Corresponding author(s): Victor Naumenko

Last updated by author(s): Nov 3, 2022

Reporting Summary

Nature Portfolio wishes to improve the reproducibility of the work that we publish. This form provides structure for consistency and transparency in reporting. For further information on Nature Portfolio policies, see our Editorial Policies and the Editorial Policy Checklist.

Statistics

For all statistical analyses, confirm that the following items are present in the figure legend, table legend, main text, or Methods section.

n/a	Confirmed	
☐	☒	The exact sample size (*n*) for each experimental group/condition, given as a discrete number and unit of measurement
☐	☒	A statement on whether measurements were taken from distinct samples or whether the same sample was measured repeatedly
☐	☒	The statistical test(s) used AND whether they are one- or two-sided<br>*Only common tests should be described solely by name; describe more complex techniques in the Methods section.*
☒	☐	A description of all covariates tested
☐	☒	A description of any assumptions or corrections, such as tests of normality and adjustment for multiple comparisons
☐	☒	A full description of the statistical parameters including central tendency (e.g. means) or other basic estimates (e.g. regression coefficient) AND variation (e.g. standard deviation) or associated estimates of uncertainty (e.g. confidence intervals)
☐	☒	For null hypothesis testing, the test statistic (e.g. *F*, *t*, *r*) with confidence intervals, effect sizes, degrees of freedom and *P* value noted<br>*Give P values as exact values whenever suitable.*
☒	☐	For Bayesian analysis, information on the choice of priors and Markov chain Monte Carlo settings
☒	☐	For hierarchical and complex designs, identification of the appropriate level for tests and full reporting of outcomes
☒	☐	Estimates of effect sizes (e.g. Cohen's *d*, Pearson's *r*), indicating how they were calculated

Our web collection on statistics for biologists contains articles on many of the points above.

Software and code

Policy information about availability of computer code

Data collection	Leica Application System X Version 1.8.1.0.13370 software was used to record IVM data. Living Image v2.5 software was used for collecting bioluminescence data. Attune Cytometer software was used for recording FC data
Data analysis	Graphpad Prism 8.0 was used for statistical analysis. FlowJo (Tree Star) and Attune Cytometer software were used for FC analysis

For manuscripts utilizing custom algorithms or software that are central to the research but not yet described in published literature, software must be made available to editors and reviewers. We strongly encourage code deposition in a community repository (e.g. GitHub). See the Nature Portfolio guidelines for submitting code & software for further information.

Data

Policy information about availability of data

All manuscripts must include a data availability statement. This statement should provide the following information, where applicable:

- Accession codes, unique identifiers, or web links for publicly available datasets
- A description of any restrictions on data availability
- For clinical datasets or third party data, please ensure that the statement adheres to our policy

The data that support the findings of this study are available within the article and its Supplementary Information and Supplementary Data 1 or from the corresponding authors on reasonable request.

Field-specific reporting

Please select the one below that is the best fit for your research. If you are not sure, read the appropriate sections before making your selection.

☒ Life sciences ☐ Behavioural & social sciences ☐ Ecological, evolutionary & environmental sciences

For a reference copy of the document with all sections, see nature.com/documents/nr-reporting-summary-flat.pdf

Life sciences study design

All studies must disclose on these points even when the disclosure is negative.

Sample size	The sample size (from n=3 to n=10 for different experimental procedures) was based on precedent of in vivo studies involving preclinical tumor models.
Data exclusions	No data was excluded.
Replication	Adequate sample sizes were used to assess reproducibility for in vivo results.
Randomization	Mice were randomly selected into the studied groups both between cages and within every single cage
Blinding	Data collection and analysis was performed blinded for all in vivo experiments.

Reporting for specific materials, systems and methods

We require information from authors about some types of materials, experimental systems and methods used in many studies. Here, indicate whether each material, system or method listed is relevant to your study. If you are not sure if a list item applies to your research, read the appropriate section before selecting a response.

Materials & experimental systems

n/a	Involved in the study
☐	☒ Antibodies
☐	☒ Eukaryotic cell lines
☒	☐ Palaeontology and archaeology
☐	☒ Animals and other organisms
☒	☐ Human research participants
☒	☐ Clinical data
☒	☐ Dual use research of concern

Methods

n/a	Involved in the study
☐	☐ ChIP-seq
☐	☒ Flow cytometry
☐	☐ MRI-based neuroimaging

Antibodies

Antibodies used	BV-421-conjugated rat anti-mouse Ly6g (clone 1A8), FITC-conjugated rat anti-mouse CD11b (M1/70), rat anti-mouse CD16/CD32 (Fc block, clone 2.4G2) were purchased from BD Biosciences Pharmingen (San Diego, CA). FITC-conjugated rat anti-mouse Ly6g (1A8), AF-488-conjugated rat anti-mouse Ly6c (HK 1.4), PE-conjugated rat anti-mouse CD169 (3D6.112), PE-conjugated rat anti-mouse F4/80 (BM8), PE-conjugated rat anti-mouse CD11b (M1/70), PE/Cy7-conjugated hamster anti-mouse CD11c (N418), PE-conjugated rat anti-mouse CD8b (YTS156.7.7), PerCP-conjugated rat anti-mouse Ly6c (HK 1.4), PerCP-conjugated rat anti-mouse CD4 (GK1.5), PE-conjugated rat anti-mouse CD19 (1D3), FITC-conjugated rat anti-mouse CD45R/B220 (RA3-6B2), APC-conjugated rat anti-mouse Ly6g (1A8), FITC-conjugated rat anti-mouse F4/80 (BM8), APC/Cy7-conjugated rat anti-mouse CD45 (30-F11), PE-conjugated rat anti-mouse Ly6g and Ly6c (R6B-8C5), PerCP-conjugated rat anti-mouse Ly6g and Ly6c (R6B-8C5) were purchased from Biolegend (San Diego, CA). Rat anti-mouse CD8a eFluor® 660 (53-6.7), PE/Cy7-conjugated rat anti-mouse CD11b (M1/70) were purchased from eBioscience (San Diego, CA). For cellular depletion, rat anti-mouse Ly6g (1A8), rat anti-mouse CD8 (YTS 169.4) were purchased from BioXcell (West Lebanon, NH). Rat anti-mouse CCR2 (MC21) antibody was kindly provided by Matthias Mack (University of Regensburg, Regensburg, Germany).
Validation	Data on validation of depleting antibodies is provided in the manuscipt. Fluorescent antibodies were tested by flow cytometry using isotype controls

Eukaryotic cell lines

Policy information about cell lines

Cell line source(s)	CT-26 LacZ cells were obtained from J. Bell (Children's Hospital of Eastern Ontario). M3-9-M cells were obtained from C. MacKall (Stanford, CA, USA).

Authentication	Cell lines were not authenticated
Mycoplasma contamination	Cells tested negative for mycoplasma
Commonly misidentified lines (See ICLAC register)	Commonly misidentified lines were not used in the study

Animals and other organisms

Policy information about studies involving animals; ARRIVE guidelines recommended for reporting animal research

Laboratory animals	Eight-week-old female BALB/c and C57bl/6 mice
Wild animals	The study did not involve wild animals
Field-collected samples	The study did not involve samples collected from the field
Ethics oversight	University of Calgary Animal Care Committee

Note that full information on the approval of the study protocol must also be provided in the manuscript.

ChIP-seq

Data deposition

☐ Confirm that both raw and final processed data have been deposited in a public database such as GEO.

☐ Confirm that you have deposited or provided access to graph files (e.g. BED files) for the called peaks.

Data access links *May remain private before publication.*	*For "Initial submission" or "Revised version" documents, provide reviewer access links. For your "Final submission" document, provide a link to the deposited data.*
Files in database submission	*Provide a list of all files available in the database submission.*
Genome browser session (e.g. UCSC)	*Provide a link to an anonymized genome browser session for "Initial submission" and "Revised version" documents only, to enable peer review. Write "no longer applicable" for "Final submission" documents.*

Methodology

Replicates	*Describe the experimental replicates, specifying number, type and replicate agreement.*
Sequencing depth	*Describe the sequencing depth for each experiment, providing the total number of reads, uniquely mapped reads, length of reads and whether they were paired- or single-end.*
Antibodies	*Describe the antibodies used for the ChIP-seq experiments; as applicable, provide supplier name, catalog number, clone name, and lot number.*
Peak calling parameters	*Specify the command line program and parameters used for read mapping and peak calling, including the ChIP, control and index files used.*
Data quality	*Describe the methods used to ensure data quality in full detail, including how many peaks are at FDR 5% and above 5-fold enrichment.*
Software	*Describe the software used to collect and analyze the ChIP-seq data. For custom code that has been deposited into a community repository, provide accession details.*

Flow Cytometry

Plots

Confirm that:

☒ The axis labels state the marker and fluorochrome used (e.g. CD4-FITC).

☒ The axis scales are clearly visible. Include numbers along axes only for bottom left plot of group (a 'group' is an analysis of identical markers).

☒ All plots are contour plots with outliers or pseudocolor plots.

☒ A numerical value for number of cells or percentage (with statistics) is provided.

Methodology

Sample preparation	Blood was obtained through cardiac puncture and collected in syringes containing 100 U heparin; red blood cells were lysed

Sample preparation	using Ammonium-Chloride-Potassium (ACK; Gibco). Tumors, spleen, and lymph nodes were harvested from euthanized animals, placed in ice-cold PBS, and homogenized by mechanical disruption. Tumor samples were additionally treated with 1 mg/ml collagenase I and 0.1 mg/ml DNase I for 30 min at 37°C and single-cell suspensions were generated using a GentleMACS (Miltenyi Biotec) followed by passage through a 70-µm nylon mesh. Following 3 washes in cold PBS, cells were blocked with anti-CD16/CD32 mAbs in FACS wash buffer (FWB; PBS, 2 % FBS, 5 mM EDTA) for 30 min at 4°C, followed by staining with fluorophore-conjugated antibodies in FWB for 30 min at 4°C. Cells were washed 3 times in FWB and analyzed
Instrument	Attune Acoustic Focusing Cytometer (Life Technologies)
Software	FlowJo (Tree Star) or Attune Cytometer software
Cell population abundance	For each sample 20 000 CD45+ cells were recorded
Gating strategy	After excluding cell debris and duplets from analysis based on FSC/SSC light scatter characteristics cells were divided into leukocytes subpopulations using antibodies listed in Antibodies section above. The boundaries between the "negative" and "positive" staining were set based on isotype controls.

☒ Tick this box to confirm that a figure exemplifying the gating strategy is provided in the Supplementary Information.

Magnetic resonance imaging

Experimental design

Design type	*Indicate task or resting state; event-related or block design.*
Design specifications	*Specify the number of blocks, trials or experimental units per session and/or subject, and specify the length of each trial or block (if trials are blocked) and interval between trials.*
Behavioral performance measures	*State number and/or type of variables recorded (e.g. correct button press, response time) and what statistics were used to establish that the subjects were performing the task as expected (e.g. mean, range, and/or standard deviation across subjects).*

Acquisition

Imaging type(s)	*Specify: functional, structural, diffusion, perfusion.*
Field strength	*Specify in Tesla*
Sequence & imaging parameters	*Specify the pulse sequence type (gradient echo, spin echo, etc.), imaging type (EPI, spiral, etc.), field of view, matrix size, slice thickness, orientation and TE/TR/flip angle.*
Area of acquisition	*State whether a whole brain scan was used OR define the area of acquisition, describing how the region was determined.*

Diffusion MRI ☐ Used ☐ Not used

Preprocessing

Preprocessing software	*Provide detail on software version and revision number and on specific parameters (model/functions, brain extraction, segmentation, smoothing kernel size, etc.).*
Normalization	*If data were normalized/standardized, describe the approach(es): specify linear or non-linear and define image types used for transformation OR indicate that data were not normalized and explain rationale for lack of normalization.*
Normalization template	*Describe the template used for normalization/transformation, specifying subject space or group standardized space (e.g. original Talairach, MNI305, ICBM152) OR indicate that the data were not normalized.*
Noise and artifact removal	*Describe your procedure(s) for artifact and structured noise removal, specifying motion parameters, tissue signals and physiological signals (heart rate, respiration).*
Volume censoring	*Define your software and/or method and criteria for volume censoring, and state the extent of such censoring.*

Statistical modeling & inference

Model type and settings	*Specify type (mass univariate, multivariate, RSA, predictive, etc.) and describe essential details of the model at the first and second levels (e.g. fixed, random or mixed effects; drift or auto-correlation).*
Effect(s) tested	*Define precise effect in terms of the task or stimulus conditions instead of psychological concepts and indicate whether ANOVA or factorial designs were used.*

Specify type of analysis: ☐ Whole brain ☐ ROI-based ☐ Both

Statistic type for inference
(See Eklund et al. 2016)

Specify voxel-wise or cluster-wise and report all relevant parameters for cluster-wise methods.

Correction

Describe the type of correction and how it is obtained for multiple comparisons (e.g. FWE, FDR, permutation or Monte Carlo).

Models & analysis

n/a	Involved in the study
☐	☐ Functional and/or effective connectivity
☐	☐ Graph analysis
☐	☐ Multivariate modeling or predictive analysis

Functional and/or effective connectivity

Report the measures of dependence used and the model details (e.g. Pearson correlation, partial correlation, mutual information).

Graph analysis

Report the dependent variable and connectivity measure, specifying weighted graph or binarized graph, subject- or group-level, and the global and/or node summaries used (e.g. clustering coefficient, efficiency, etc.).

Multivariate modeling and predictive analysis

Specify independent variables, features extraction and dimension reduction, model, training and evaluation metrics.
